# Evaluation of Depression, Anxiety, and Stress in Adolescents and Young Adults with Auditory Neuropathy Spectrum Disorder

**DOI:** 10.1155/2016/4378269

**Published:** 2016-08-08

**Authors:** Prashanth Prabhu

**Affiliations:** All India Institute of Speech and Hearing, Mysore 570 006, India

## Abstract

The aim of the present study was to determine the severity of stress, anxiety, and depression using Depression Anxiety Stress Scales (DASS) in adolescents and young adults with auditory neuropathy spectrum disorder (ANSD). DASS was administered to 20 individuals with auditory neuropathy spectrum disorder. The effect of gender on severity of anxiety, stress, and depression on DASS scores was determined. It was attempted to determine the correlation of severity of anxiety, stress, and depression with the reported onset of the problem, degree of hearing loss, and speech identification scores. The results of the study showed that individuals with ANSD had a moderate degree of depression and anxiety. The results also showed that the symptoms were more seen in females than in males. Correlation analysis revealed that DASS scores correlated with the reported onset of condition and speech identification scores (SIS) and the degree of hearing loss showed no correlation. The study concludes that individuals with ANSD experience depression and anxiety and this could be because of the inadequate management options available for individuals with ANSD. Thus, there is a need to develop appropriate management strategies for individuals with ANSD and provide appropriate referral for management of psychological issues.

## 1. Introduction

Auditory neuropathy spectrum disorder (ANSD) is defined as a disorder in which a patient has normal outer hair cells functioning (represented by normal otoacoustic emissions/cochlear microphonics) and an absent/abnormal auditory brainstem response (ABR) [1–5]. The prevalence rate of ANSD varies from 1% [6] to 10% in schools for the deaf [7–9] and between 10% in newborns [10] and 40% in hearing-impaired NICU patients [11]. The onset of ANSD symptoms tends to fall into two different age groups. The symptoms may be exhibited in infancy and childhood or the symptoms may develop in adolescence or early adulthood [1, 12, 13]. Majority of reports from the western population suggest that only one out of four ANSD patients are over the age of 10 years [1, 12, 14]. In contrast, reports from the Indian population have shown that symptoms onset was majorly in adolescence (16 to 25 years) [13, 15, 16].

The management of individuals with ANSD is a challenge for clinical audiologists because of the uncertainty in etiology and pathophysiology. A number of researchers have demonstrated that, in adults and children with ANSD, acceptance of amplification is universally poor with reports ranging from little or no benefit to detrimental effects [2, 3]. In contrast, few other investigators have demonstrated that a group of patients do benefit from amplification but the amount of benefit is not comparable with those observed in individuals with cochlear hearing loss [17, 18]. Cochlear implants (CI) have been found to be useful in some individuals with ANSD [19, 20]. However, recent investigators have demonstrated that not all subjects with ANSD benefit from cochlear implants and the benefit seems to depend on the site of the lesion [18]. Berlin et al. [1] summarized the assessment and management of 260 patients with ANSD. They reported that approximately 15% got benefit from hearing aids and speech and language improvement was reported in 85% of patients who had implantation. Hence, cochlear implants have proven to be the most accepted management option for individuals with ANSD. However, in a developing country like India, it is very difficult for patients to afford CI as the majority of patients with late onset ANSD are of low socioeconomic status [15]. Thus, hearing aids are the next viable option available for management of individuals with ANSD. Narne et al. [21] studied hearing aid benefit in 128 individuals with ANSD in India and reported that 89 patients with ANSD did not get benefit from hearing aids and only 26 of them were getting benefit from hearing aids in functional use whereas the other 13 had limited usefulness of the hearing aid (only for sound detection and awareness). Thus, at present, the management options for late onset ANSD are limited especially in a developing country like India that cannot afford costly CI and frequency modulation (FM) devices.

Thus, late onset ANSD can be a quite debilitating condition as the clients are perfectly normal till adolescence and suddenly exhibit auditory symptoms. This leads to poor communication among the peer group and social isolation and decline in academic performance. The problem becomes more serious as there are no definite management options for individuals with late onset ANSD. Most of the clients are advised to use speech reading and manage without hearing aids, as the hearing aids may worsen the performance and can lead to tolerance problems. All these factors can lead to psychological issues such as stress, depression, and anxiety in individuals with late onset ANSD. It has been well reported in the literature that children with cochlear hearing loss experience emotional difficulties [22, 23]. Children and adolescents with cochlear hearing loss score poorer than normal hearing peers on measures of emotional and behavioral difficulties [22]. However, there are no studies reported in the literature assessing the psychological problems faced by children and adolescents with ANSD.

There are several validated tests and scales reported in the literature which can be used to determine anxiety, stress, and depression such as Goldberg screen for depression [24], Composite International Diagnostic Interview [25], PRIME-MD [26], and Depression Anxiety Stress Scales (DASS) [27]. DASS is an excellent tool which corresponds to the tripartite model of anxiety and depression [28]. Therefore, the structure of the DASS seems to support the view that both anxiety disorders and depression need to be distinguished from adjustment disorders in spite of their similarities. The psychometric properties of this instrument appear to be sound enough to be applied to both healthy and psychiatric populations. The internal consistency of the three subscales ranged from 0.81 to 0.97 along with good convergent and divergent validity [29–32].

The studies on auditory neuropathy spectrum disorder have mainly focused on audiological difficulties of the condition. There is very limited research which has assessed functional aspects like social and emotional consequences because of ANSD. There are anecdotal references about depressive symptoms, suicidal tendencies, anxiety, and stress in individuals with late onset ANSD. However, there are no systematic studies reported in the literature which assess the psychological issues in individuals with ANSD. Thus, the present study was designed with an objective to assess stress, anxiety, and depression in individuals with ANSD using DASS. The aims of the study were to determine the severity of stress, anxiety, and depression using DASS in adolescents and young adults with late onset ANSD. It was also attempted to determine the effect of gender and the correlation of severity of anxiety, stress, and depression with the onset of the problem, degree of hearing loss, and speech identification scores.

## 2. Materials and Methods

The study comprised 20 individuals (8 males and 12 females) who were diagnosed as having ANSD. The mean age of ANSD group for males and females was 16.6 and 21.4, respectively. All the participants reported that they have no audiological symptoms till 12 years of age and the reported onset of condition ranged from 12 years to 17 years. All the participants had normal speech and language assessed through informal screening method. ANSD was diagnosed based on the criteria recommended by Starr et al. [14]. They are preserved cochlear amplification, reflected by the presence of transient evoked otoacoustic emissions and/or cochlear microphonics; abnormal auditory nerve responses as indicated by absent or severely abnormal auditory brainstem responses; and normal otologic and tympanometric findings with absent acoustic reflexes. A detailed neurological examination was carried out on all individuals to rule out any space occupying lesion by a neurologist with a detailed clinical neurologic examination that also included the result from radiologic investigations such as CT/MRI.

### 2.1. Procedure

All the individuals considered for the study were tested with calibrated audiometers in sound treated rooms. Pure tone air conduction (AC) and bone conduction (BC) thresholds were estimated using modified Hughson and Westlake procedure [33]. AC thresholds were obtained for pure tones from 250 Hz to 8 kHz and BC thresholds from 250 Hz to 4 kHz in octave frequencies. A two-channel diagnostic audiometer OB-922 coupled with impedance matched TDH 39 earphones and a bone vibrator (RadioEar B-71) was used to obtain air conduction and bone conduction pure tone thresholds and speech identification scores. Unaided speech identification scores were obtained for phonemically balanced words developed for adults in Kannada by Yathiraj and Vijayalakshmi [34]. Recorded word lists were routed from a PC through a two-channel diagnostic audiometer (OB-922) to TDH 39 headphones at 40 dB SL (re: SRT). An immittance meter from Grason Stadler Inc., TympStar (GSI-TS), was used for immittance testing. Each ear of the participant was tested to obtain tympanogram and acoustic reflexes for a probe tone frequency of 226 Hz. Acoustic reflexes were measured using 500, 1000, 2000, and 4000 Hz pure tones, presented to both ipsilateral and contralateral ears. Otodynamics ILO v.6 OAE analyzer was used to obtain transient evoked otoacoustic emissions (TEOAEs). After ensuring probe fit, TEOAEs were measured for nonlinear click trains presented at 80 dB peak equivalent SPL. Waveform reproducibility of more than 50% [35] and an overall signal-to-noise ratio of more than 3 dB SPL [36] at least at two frequency bands were required to be considered as presence of TEOAEs.

Biologic Navigator Pro (Bio-logic, Mundelein, IL) AEP system with ER 3A insert earphones was used to record ABR. ABR was recorded with the clients seated on a reclining chair. The skin surfaces on the two mastoids and forehead were cleaned with a skin abrasive. Gold cup electrodes were used to record responses. The electrodes were placed with the help of skin conduction paste and surgical plaster was used to hold the electrodes tightly on the respective places. Absolute electrode impedance was maintained below 5 kΩ with interelectrode impedance below 2 kΩ. Before starting the recording, participants were instructed to relax and refrain from extraneous body movements to minimize artifacts. Single channel recordings were obtained with inverting electrode on the test ear mastoid (M2/M1), noninverting electrode on the high forehead (Fz), and ground electrode on the nontest ear mastoid (M1/M2). Click evoked ABR was recorded twice and replicated for 100 *µ*sec click stimuli delivered at a repetition rate of 11.1 clicks/second at 90 dB nHL. The recording was obtained for a total of 1500 sweeps and a filter setting of 100 Hz to 3000 Hz was used.

### 2.2. Administration of DASS

DASS is a 42-item questionnaire which includes three self-report scales designed to measure the negative emotional states of depression, anxiety, and stress. Each of the three scales contains 14 items, divided into subscales of 2–5 items with similar content. The depression scale assesses dysphoria, hopelessness, devaluation of life, self-deprecation, lack of interest/involvement, anhedonia, and lack of inertia. The anxiety scale assesses autonomic arousal, skeletal muscle effects, situational anxiety, and subjective experience of anxious affect. The stress scale (items) is sensitive to levels of chronic nonspecific arousal. It assesses difficulty relaxing, nervous arousal, and being easily upset/agitated, irritable/overreactive, and impatient. Respondents are asked to use 4-point severity/frequency scales to rate the extent to which they have experienced each state over the past week. DASS was administered to all the participants of the study.

### 2.3. Ethical Considerations

In the present study, all the testing procedures done were using a noninvasive technique adhering to conditions of ethical approval committee of the institute and complied with the Declaration of Helsinki. All the test procedures were explained to the patients and their family members before testing and informed consent has been taken from the patients and their family members for participating in the study.

## 3. Results

**Figure 1:**
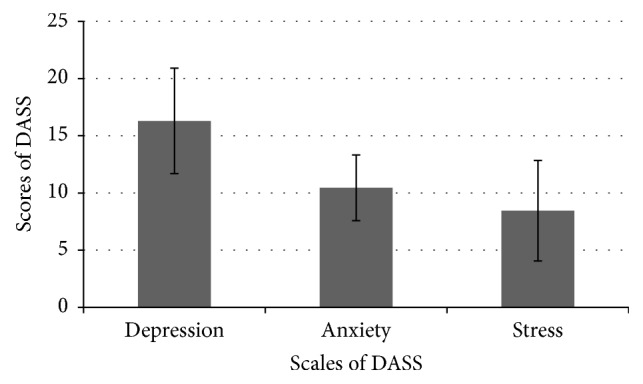
The mean and SD of the subscales of DASS in the participants of the study.

The result of the study shows that individuals with ANSD do experience depression, anxiety, and stress because of the condition. The mean DASS score obtained for depression subscale was 16.3 (SD = 4.6), for anxiety subscale was 10.45 (SD = 2.88), and for stress subscale was 8.45 (SD = 4.39). These results for all the three subscales are depicted graphically in Figure 1. The results were compared with the severity rating of depression, anxiety, and stress. Figure 1 shows that depression and anxiety were of moderate degree and stress level was normal. The result shows that all the participants had emotional problems associated with the condition of ANSD.

**Figure 2:**
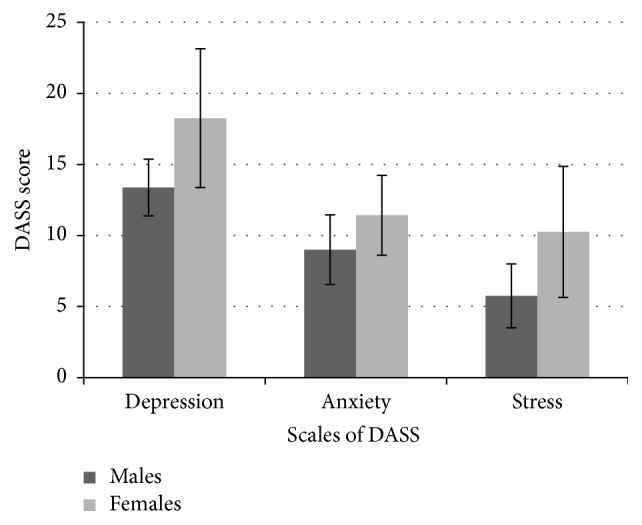
The mean and SD of DASS scores for the three subscales across gender.

The result was also analyzed in terms of effect of gender on the DASS scores obtained for all the three subscales. The result shows that the mean scores for all the three subscales were higher for females compared to males. The mean depression, anxiety, and stress scores for males were 13.37 (SD = 1.9), 9 (SD = 2.44), and 5.75 (SD = 2.25), respectively. The mean depression, anxiety, and stress scores for females were 18.25 (SD = 4.88), 11.42 (SD = 2.81), and 10.25 (SD = 4.6), respectively. The results obtained are depicted graphically in Figure 2. Mann-Whitney *U* test was carried out to check whether the scores obtained across gender were significantly different or not for all the three subscales. There was a significant difference for depression (*p* < 0.01) and stress (*p* < 0.05) between males and females with ANSD. However, there was no significant difference (*p* > 0.05) for anxiety between males and females with ANSD considered for the study.

**Figure 3:**
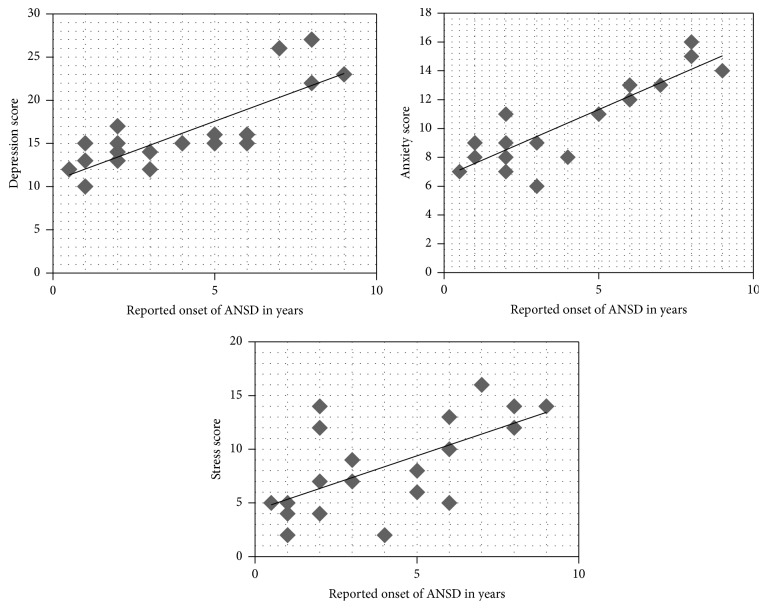
Scatter plots of correlation between the reported onset of ANSD in years and the different scales of DASS.

It was also attempted to determine the correlation between the reported onset of ANSD, pure tone average, and speech identification scores with DASS scores. Nonparametric Spearman's rank correlation was used for the statistical analysis. The results show that there was a high positive correlation for depression (*ρ* = 0.79, *p* < 0.01) and anxiety (*ρ* = 0.817, *p* < 0.01) and a moderate positive correlation for stress (*ρ* = 0.631, *p* < 0.01) with respect to the reported onset of ANSD. The results on correlation of age of reported onset and DASS scores are shown graphically using scatter plots in Figure 3.

**Figure 4:**
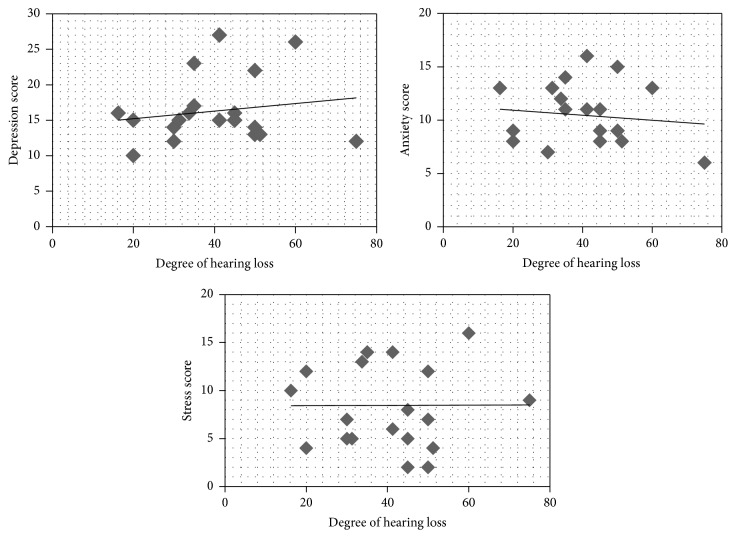
Scatter plots of correlation between the degree of hearing loss and the different scales of DASS.

**Figure 5:**
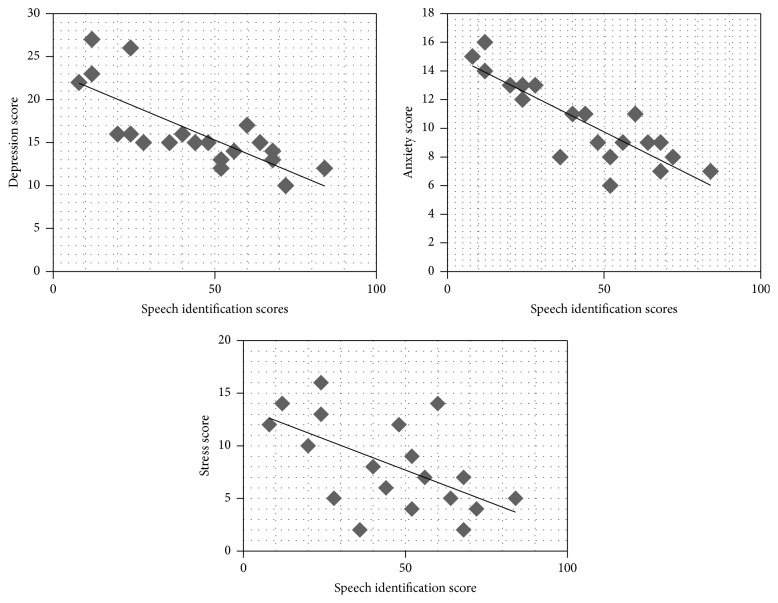
Scatter plots of correlation between the average speech identification scores and the different scales of DASS.

The correlation between speech pure tone average (PTA) and DASS scores was analyzed. The result shows that there was no correlation for depression (*ρ* = 0.032, *p* > 0.05), anxiety (*ρ* = 0.04, *p* > 0.05), and stress (*ρ* = 0.03, *p* > 0.05) with PTA. The results on correlation of PTA and DASS scores are shown graphically using scatter plots in Figure 4. In addition, the correlation between speech identifications scores (SIS) and DASS scores was analyzed. The result shows that there is a high negative correlation between SIS and depression (*ρ* = 0.806, *p* < 0.01) and anxiety (*ρ* = 0.822, *p* < 0.01) and a moderate negative correlation with stress (*ρ* = 0.584, *p* < 0.05) scores. The results on correlation of SIS and DASS scores are shown in Figure 5.

## 4. Discussion

The result of the study shows that all the clients with auditory neuropathy spectrum disorder considered for the study experienced anxiety and depression. The severity of the anxiety and depression in individuals with ANSD was of moderate degree. This could be because of the limited management options available for management of individuals with late onset ANSD. The individuals with cochlear hearing loss also experience emotional difficulties [22, 23]. It is reported in the literature that children and adolescents with cochlear hearing loss score poorer than normal hearing peers on measures of emotional and behavioral difficulties [22]. However, with appropriate use of hearing aids, emotional difficulties are reported to reduce in individuals with cochlear hearing loss [37]. In the present study, hearing aids were not beneficial for all the participants considered for the study. Only four participants were using hearing aid with limited benefit. The literature also shows that hearing aids are reported to provide limited benefit in children with ANSD in majority of the studies [1, 2, 38]. Berlin et al. [1] in a study on a large population with ANSD reported that hearing aids were beneficial in only 11 of the 94 patients in their study. Jijo and Yathiraj [39] studied hearing aid benefit in 120 clients diagnosed as having late onset auditory neuropathy spectrum disorder. They reported that 70% of the individuals with ANSD did not get benefit from hearing aids. Narne et al. [21] also studied 128 individuals with late onset ANSD and reported that hearing aids were not beneficial in 89 individuals in their study and were useful for speech understanding in 26 and were useful only for awareness in 13 of them. All these 89 individuals who did not get benefit from hearing aids were counseled regarding the other management options such as FM devices and cochlear implants and were advised to use speech reading [21]. In the current Indian scenario, cochlear implants and FM devices are expensive and not affordable for majority of the individuals. Thus, they are forced to learn speech reading on which they are trained and to just manage their life without understanding speech in auditory mode. This can have a debilitating effect on the lives of individuals with ANSD and can lead to psychological issues. The increased depression and anxiety in their lives thus could be because of the lack of hope in their lives that they cannot be treated. Hence, the lack of appropriate management options for individuals with late onset ANSD can lead to anxiousness and depression in individuals with ANSD.

The result of the study showed that the scores for depression, stress, and anxiety were more for females than for males. This is in consensus with studies on depression reporting that depression occurs approximately twice as often in women compared to men [40]. Depression and anxiety are reported to be the most common comorbid disorders and a significant gender difference exists in the rate of comorbidity [41]. The family structure in majority of the families in India needs women to communicate more with the children they are raising. In addition, majority of rural India still follows a patriarch system where men go for work and women run the family. Thus, the increased need for effective communication by women could have led to poorer scores in females than in males. The result of the study also shows a high positive correlation for depression and anxiety and moderate positive correlation for stress with respect to reported onset of ANSD. Thus, when the duration of the condition was longer, the severity of depression and anxiety increased drastically. This is expected since they are not getting appropriate management and the condition may progress over time in individuals with ANSD. In addition, ignorance regarding the exact reason for their hearing difficulties could have also led to these symptoms. Thus, early diagnosis and management of ANSD could lead to reduction in symptoms of depression, anxiety, and stress. Hence, it is stressed that there is an urgent need for research on understanding the physiology of ANSD in detail and designing an appropriate management strategy which is different from the traditional approaches to help individuals with ANSD.

The result of the study also shows that there was no correlation between pure tone thresholds and DASS scores. This suggests that irrespective of the degree of hearing loss they may experience symptoms of depression, anxiety, and stress. Thus, degree of hearing loss should not be used as the criteria to determine the psychological effects as the degree of loss may fluctuate in individuals with ANSD and it is not a predictor of the extent of the condition [1, 38, 42]. The result also shows that there is a high negative correlation between SIS and depression and anxiety and moderate negative correlation for stress. The decreased speech identification abilities in individuals with ANSD affect their daily lives functioning and thus can lead to depression and anxiety symptoms. Thus, the symptoms of depression, anxiety, and stress increase with decreased SIS and duration of the disorder and are not related to the degree of hearing loss. However, these emotional and psychological difficulties faced by individuals with ANSD are not given importance in the rehabilitation. Hence, the study highlights the need for psychological counseling and appropriate measures for management of depression and anxiety in individuals with ANSD.

### 4.1. Limitations of the Study

The study needs to be further carried out on a larger group with ANSD for better generalization of the results. Some of the individuals with reported late onset ANSD could have congenital ANSD and might have reported late because of lack of universal newborn hearing/school screening available in India. In addition, poor socioeconomic status could have affected an early intervention. Thus, it is difficult to generalize the results only to late onset ANSD. Low socioeconomic status can lead to emotional problems even in individuals with normal hearing [43]. But, with ANSD and low socioeconomic status, they are expected to have more problems. However, it would be advised to compare DASS scores of ANSD group and normal hearing peers with low socioeconomic status. The results need to be compared with age matched cochlear hearing loss group, early onset ANSD group, and ANSD group who are successfully rehabilitated with hearing aids or cochlear implants. This would justify the claims of the study which suggest that inappropriate management strategies, late onset, and poor speech understanding are the possible reasons for the psychological problems seen in individuals with ANSD.

## 5. Conclusions

The study attempted to evaluate depression, anxiety, and stress in a group of individuals diagnosed with auditory neuropathy spectrum disorder using DASS. The study tried to assess the emotional disturbances faced by these individuals who do not get benefit from hearing aids and cannot afford cochlear implants or FM devices. The results of the study showed that individuals with ANSD had a moderate degree of depression and anxiety. The results also showed that the symptoms were more seen in females than in males. The correlation analysis revealed that DASS scores correlated with the reported onset of condition and SIS and the degree of hearing loss showed no correlation. Hence, the study explores the notion that individuals with ANSD do experience depression and anxiety and this could be because of the inadequate management options available for individuals with late onset ANSD. Thus, there is a need to refer clients with ANSD also for a detailed psychological assessment and possible rehabilitation.
